# Gleanings from the German

**Published:** 1874-10-15

**Authors:** E. F. Dœring


### Translations.

### GLEANINGS FROM THE GERMAN.

*Collated by Dr. E. J. Dœring.*

### THE INFLUENCE OF DEW ON THE ORIGIN OF MALARIAL FEVERS.

DR. AYR, of Rome, Italy, sums up his experience regarding the influence which dew exerts in the pathogenesis of malarial fever, and the protective influence of woolen, cotton and linen clothing, as follows:

1st. The theory of an inorganic volatile miasm, which is condensed in the dew and then breathed in with the air, is without scientific proof.

2d. On the other hand it must be accepted that the miasm is organic, and consists of mycrophytes floating in the air.

3d. The dew imbibes the miasm, and thus purifies the atmosphere of noxious poisons. But it is only after dew and fog have separated, fallen and evaporated, that the malarial poison condenses on the soil. This occurs most generally at sunrise and while the wind is blowing, at which time the air inspired becomes dangerous and poisonous.

4th. Dew is only an occasional cause of the origin of malarial fevers, through the rapid hygrothermic change and its influence upon the organism.

5th. To avoid therefore these injurious hygrothermic influences, it is recommended in malarial districts to wear next to the skin linen, and not cotton or woolen shirts, as the former are less hygrometric, allowing the air to permeate, but without aqueous vapor, and without malarial sporules.

### THERAPEUTIC USES OF NITRITE OF AMYL.

In a recent number of the "Deutches Arch. f. Klin. Med.," is an interesting article by Dr. Fuckel, on the above subject. He has obtained good results with the nitrite in hemicrania, likewise in cardialgia. In the latter affection the results were especially beneficial. Dr. F. used it in 13 different cases with immediate success. A few seconds after the inhalation the pain disappeared at once, even in the most aggravated cases. In a few patients the pain returned after half an hour or later, but subsided again after a renewed inhalation. The same drug proved further successful in the treatment of neuralgia accompanying menstruation. Dr. F. tried it in 6 cases, in all of which the remedy either caused an immediate cessation of the pain, or a great amelioration.

The dose employed for inhalation consisted of three drops, repeated if necessary.

### CARBOLIC ACID AS AN ANTIPHLOGISTIC.

Dr. Hagen reports in the "Aerzt. Intelligens Blatt, No. 32," several cases, viz.: Phlegmonous inflammation of the hand, croupous pneumonia, pharyngitis and tonsilitis, croup, etc., which he treated successfully by injections of a two per cent. solution of carbolic acid. After the first injections the pain diminished and the temperature was reduced. The locality for the injection corresponded to the seat of the inflammation, e. g., for croup, in the region of the crocoid cartilage, etc. Dr. Kunze likewise reports a few cases of pneumonia, and articular rheumatism treated with good results by means of these injections.

PROF. ESMARCH'S METHOD OF BLOODLESS OPERATION.

Dr. Duus in Kiel has written an essay on the subject of artificial anæmia in surgical operations, giving the results obtained by Prof. Esmarch in the City Hospital in Kiel. Two hundred and twenty-six operations were performed by the Professor during the period from February, 1873, till June, 1874.

Out of the above number only 15 patients died, and but 7 were discharged not improved. Cases of paralysis following the use of the elastic bandage, as reported by Prof. V. Langenbeck, were not observed. Nor did any cases of septicæmia (through possible introduction of septic matter from pressure of the bandages), nor gangrene of the flaps after amputation occur. Secondary hæmorrhage took place a few times, but was so slight as not to require any interference.

In conclusion the author presents the following valuable statistical table, showing the comparative results obtained by different operators.

Table showing mortality after amputations and disarticulations of larger joints as given by different authorities.

| OPERATOR. | No. of Cases. | Deaths. | Mortality. per cent. |
|---|---|---|---|
| Esmarch | 34 | 4 | 11.8 |
| Lister | 76 | 20 | 26.3 |
| Erichsen | 80 | 21 | 26.3 |
| Volkmann | 46 | 13 | 28.2 |

ENEMATA OF BROMIDE OF POTASSIUM IN OBSTINATE VOMITING.—Dr. Girabetti has obtained the very best results from the administration of enemata of bromide of potassium, in doses of from one-half to two drachms, in cases of obstinate vomiting attending the pregnant state. The same drug, also administered in enemata, has been very successful in the hands of Dr. Laborde, of Paris, in obstinate vomiting, connected with disease of the stomach, liver and intestines.

DEEP INJECTION OF CHLOROFORM FOR THE RELIEF OF TIC DOULOUREUX.—Dr. Roberts Bartholow communicates to *The Practitioner* (June, 1874), an account of several cases of this painful affection treated successfully by hypodermic injections of chloroform.

The infra-orbital branch of the nerve was the seat of the *tic* in the cases reported, and Dr. B.'s operation consisted in passing the needle under the upper lip in the direction of and near to the infra-orbital foramen, and then injecting from ten to twenty minims of pure chloroform. Considerable pain at first ensues, followed by a feeling of numbness and anæsthesia of the parts into which the chloroform diffuses. A puffy swelling quickly forms at the site of the injection, and an induration which lasts for several days follows. One very severe case operated upon in this manner gained relief from one injection covering a period of months.—*Phil. Med. Times.*

